# Blood substitutes: Basic science, translational studies and clinical trials

**DOI:** 10.3389/fmedt.2022.989829

**Published:** 2022-08-18

**Authors:** Jonathan S. Jahr

**Affiliations:** Department of Anesthesiology and Perioperative Medicine, David Geffen School of Medicine, University of California, Los Angeles, Los Angeles, CA, United States

**Keywords:** blood substitutes, artificial oxygen carriers, Hemopure, HBOC-201, hemoglobin

## Introduction

According to recent reports, the yearly mortality rate is 1.9 million worldwide from hemorrhage (1). This stunning statistic has been slightly improved from the estimated 5 million deaths worldwide each year from earlier reports (2); however, compared with COVID-19, this extreme death rate is continuing, yet relatively little research and productivity has been seen over the last 5 decades regarding improving survival, as the treatment of acute anemia is relatively simple as compared with deriving new vaccines, etc. for a pandemic.

### Setting the stage for need of blood substitutes

This work will describe the nature of the problem, hemorrhage, work that has been performed for almost 50 years to ameliorate hemorrhage and acute anemia, with treatments other than transfusion of erythrocytes. There follows a brief history of hemoglobin-based oxygen carriers (HBOCs); erythrocytes may not be available in emergency situations and where patients refuse transfusion and where blood is not an option (3). Studies performed in this area will be documented in three sections: Basic Science, Translational Studies and Clinical Trials.

### Hemorrhage

When accident victims are bleeding to death, there have been few medical treatment options to save their lives until they can be rescued and transported to a hospital, despite new campaigns, including use of tranexamic acid as an antifbrinolytic (4).

Therefore, work that proposes to develop a new type of fluid, with multiple medical effects, that is designed to improve survival from first-aid treatment by a healthcare professional is warranted. It will include fluids, oxygenating capability (like erythrocytes), blood clotting capability and other essential factors to improve survival—a revolutionary concept for the improvement of current hemorrhagic trauma medical care.

### Transfusion medicine and current standard of care

Blood transfusion, including use of erythrocytes, plasma, and platelets, is currently the most effective therapy for hemorrhage but often not immediately available during out of-hospital settings where most traumatic injuries occur (remote sites of injury or battlefields). Current standard prehospital therapy for hemorrhage is infusion of isotonic crystalloid solutions (less commonly, colloids and, rarely in civilian and military trauma, blood, and blood products) (5). However, the large volumes of crystalloids often required to maintain BP in these patients may cause fluid overload and dilution of red cells (anemia), hemostatic and other beneficial mediators in blood. In addition, crystalloid volume expanders neither carry the oxygen that is essential to maintain vital organ functions nor the coagulation factors that prevent further blood loss.

A recent Cochrane project (6) outlines a protocol whose objective is to assess the effects and safety of blood product transfusion strategies started during the first day post injury for trauma patients of all ages with major bleeding. Clearly, the need to understand and improve the standard of care is warranted, possibly including blood substitutes and/or oxygen carriers.

All recent iterations of guidelines for resuscitation following hemorrhage and trauma acknowledge the challenge of providing oxygen carrying capacity early on in the injury and that later coagulation concerns become paramount to resolution for survival to occur (7).

NICE Pathways have been constructed for hemorrhage in the hospital setting^1^, which also indicate that the need for rapid oxygen carrying delivery is key to survival and later coagulation concerns are vital. However, without a blood substitute/oxygen therapeutic, such delivery to ischemic tissues is difficult and sometimes impossible without ECMO or cardiopulmonary bypass, given the need to have pulsatile blood flow in order for erythrocytes to offload oxygen in the capillaries. It is conceivable that oxygen therapeutics, depending on design, may deliver oxygen in non-pulsatile situations, such as cardiopulmonary arrest with resuscitation and even chest compressions.

Recent trials, such as PAMPER, have attempted to improve this but only with currently available blood products (5). Therefore, development of a “blood substitute? that is efficacious and without risk for management of hemorrhage, including coagulopathy, has been a long-standing goal which remains unfulfilled (3).

### Traumatic hemorrhagic shock

Traumatic hemorrhagic shock (THS), as compared with surgical hemorrhage, is a high mortality, multiple pathophysiology condition, including hypovolemic anemia, coagulopathy, inflammation, and infection due to open wounds and damaged blood vessels. Erythrocyte, plasma, and platelet administration is currently the most effective therapy for traumatic hemorrhagic shock, but it is rarely available for out-of-hospital settings where most traumatic injuries occur. Current standard prehospital treatment for THS is infusion of isotonic crystalloid solution administered by the first responder healthcare professionals. Therefore, crystalloids formulated as hypertonic solutions (3–7%) have been proposed to be more effective in the resuscitation of hemorrhagic patients as they may permit “small volume” resuscitation thus preventing fluid overload and secondary hemorrhage that may occur with conventional isotonic resuscitation that typically requires 2–3 times the volume (8). In addition, small volume hypertonic solutions may provide anti-inflammatory effects; however, recent trials have suggested that these are ineffective (8). Although crystalloids may be effective in blood volume expansion, they do not transport the oxygen critical for vital organ function and survival. In addition, trauma victims often present with coagulopathy that causes uncontrollable bleeding. Intravenous use of recombinant human coagulation factor VIIa in hospitalized trauma patients have been studied and appears efficacious in soldiers wounded in the battlefield during the Iraq war (9). In the Iraq war experience, early use of factor VIIa reduced blood transfusions by 20%. Other trials have indicated promise, though no better outcomes (10).

### Brief history of hemoglobin-based oxygen carriers (HBOC)

The recent review on the topic is used as a reference (3). It reviews the science to date as of its composition and highlights 11 of my significant publications in this area (11–21).

Additionally, it provides the reader with an update on the newest of products currently under study. Additionally, the review sheds light on the worldwide need for these products when blood is either unavailable or not an option, whether for personal reasons, unavailability or for incompatibility.

Hemoglobin-based oxygen carriers were created as early as 1934, when Amberson purified bovine hemoglobin and infused it into study feline models and summarized in 1949, when he described purified human hemoglobin being infused into anemic parturients with hemorrhage after childbirth (22). Development continued when the US Army manufactured a tetrameric cross-linked hemoglobin (α-α cross-linked hemoglobin) which later was produced by the Baxter Corporation (Deerfield, IL), as 2,3-diaspirin cross-linked hemoglobin (HemAssist) (23). However, it failed in human studies because of decreased cellular perfusion and increased morbidity and mortality (3). During a resurgence of activity in the mid-1980s, manufacturers developed second- generation HBOCs, including Biopure Corp. (Cambridge, MA) with HBOC-200 (Oxyglobin, approved by the FDA and the European Union for canine anemia in 1997 and 1998, respectively); Hemoglobin-glutamer-201 (Hemopure, approved in South Africa for treatment of human anemia in 2001 and Russia in 2006); PolyHeme by Northfield Laboratories (Evanston, IL); Hemolink by Hemosol, Inc. (Mississauga, ON, Canada) (24). Several studies critically evaluated these products both in animal studies and human trials, documenting successful outcomes being met in Phase I, II and III trials (25, 26).

### Allogeneic blood transfusion risks

Blood transfusions are amongst the most common hospital procedures and have been in practice since 1795. Despite their prevalence, risks that are related to blood transfusions are most commonly: iron overload, TRALI (transfusion related acute lung injury) and resultant death in significant proportions (27). Other risks which, thanks to updated practice, have been mitigated, include transmitted disease (Chagas disease, HIV, Hepatitis C, malaria etc.), compatibility complications and TRALI (28). Blood product ease of access including the risks have directed the requirement for an oxygen carrier that is both safe and effective. A suitable blood substitute would eliminate the need for cross matching, reduce risk of pathogen transmission, increase availability in remote regions and be storable for longer periods of time. Much work has been done to create blood substitutes. The first involved removing the erythrocyte (RBC) coat and infusing stroma-free hemoglobin (SFH). However, infusion of SFH imitates extreme hemolysis, leading to jaundice, renal failure and death (11). To increase stability and avoid hemolysis, hemoglobin-based oxygen carriers (HBOC) use purified human, animal, or recombinant hemoglobin (Hb) in a cell-free preparation (11). Early generation HBOCs had adverse events, including renal failure, scavenging of nitric oxide (NO) causing vasoconstriction and oxidation causing methemoglobinemia (29).

These adverse effects are due to tetramers of hemoglobin being cleaved into dimers. Alleviating the adverse effects, specifically decreasing the severity of vasoconstriction, has led to several modifications such as cross-linkage, polymerization and encapsulation with polyethylene glycol (PEGylation), which will all be discussed (30).

### Advances in second generation HBOCs and beyond

Stroma-free hemoglobin were produced by either ultrafiltration or crystallization. The ultrafiltration method yielded SFH free of vasocontraction and contractility-depressant effects when tested on an *ex vivo* perfused heart (31). Although the adverse effects described above prevented use of SFH, results were useful in showing ultrafiltration should be the preferred method of production. Improving upon SFH, it was hypothesized that crosslinking might help in reducing the adverse effects. The concepts were evaluated with diaspirin-crosslinked Hb (DCLHb), known as HemAssist. Due to increased mortality, the studies on this product were terminated. The patients experienced major vasoconstrictive adverse events (23).

Following the failure of HemAssist, a second generation of HBOCs was created. The first was Hemolink, (24). Hemolink is a raffinose crosslinked HBOC prepared from outdated human erythrocytes (24).

The next HBOC, Polyheme, was a glutaraldehyde polymerized human Hb from outdated RBCs and is primarily used as a resuscitation fluid (25). The product was discontinued after Phase III following negative results of imbalance in mortality (32). It is unclear if the poor outcomes resulted from the product or protocol of the trials. There were also some ethical issues raised in the conduct of trauma trials having to do with lack of subject consent and community assent issues (33).

The final product of the second generation of HBOCs was Hemopure (HBOC-201) and made from purified bovine hemoglobin cross-linked and then polymerized with glutaraldehyde. Hemopure does not contain any cell blood membrane antigens, so may be tolerated by all blood types (16). Its benefit arises from its ability to maintain O 2 delivery to ischemic tissues during anemia or low blood flow.

Following the setbacks of the second generation HBOCs, researchers focused on a product that would avoid the toxic chemical and biophysical effects demonstrated by the crosslinked/polymerized HBOCs. Instead of producing a substitute for blood transfusions, researchers began developing a product for situations in which blood is not available for transfusion or in situations in which a blood transfusion is not possible due to health or religious objections, for example, Jehovah's Witness. Further work showed that an oxygen-binding agent may be more valuable in preventing or treating ischemia-related morbidity, thereby reducing mortality (34).

The first product to come from this new, shifted thinking was Hemospan/MP4 (Sangart, San Diego, CA). Hemoglobin derived from outdated human blood was modified with Maleimide-Polyethylene Glycol and regarded as a plasma expander due to its purported vasodilatory effects, which was in stark contrast to its vasoconstrictive predecessors.

MP4 showed promise in its Phase I study when, upon administration to healthy patients, it did not induce hypertension or cause any gastrointestinal side effects, unlike other HBOCs (35). However, in further Phase II trials, MP4 administration did cause vasoconstriction due to release of un-crosslinked free hemoglobin and, therefore, was not successful in alleviating issues from earlier HBOCs (3).

### Update on newer/newest products that have been studied in humans

Current development with new concepts in products which change former generations HBOC characteristics of lowered hemoglobin (from 10–13 g/dL to 4–6 g/dL) and oxyhemoglobin dissociation shifting the curve to the left from the right (p50 30–40 mmHg to 6 mmHg) (OxyVita and MP4) (3, 17). These strategies for improved function remain to be validated and verified independently. The newest generation HBOCs to be evaluated in human trials, Saguinate (Prolong Pharmaceuticals, Piscataway, NJ) has similar pharmacologic profiles, and some successful trials have been published (3). A marine worm derived HBOC, from Hemarina (Morlaix, Brittany, France) has also been evaluated in a few human trials and also for use in pulmonary failure related to COVID-19 (3, 36).

## Clinical trials

Multiple clinical trials have been executed on HBOCs, to evaluate their safety and efficacy in various disease states. The area most studied has been as oxygen carriers to facilitate transport to hypoxemic and ischemic tissues and hopefully resume homeostasis within the organism and protect survival. The following two studies are examples of clinical trials that exemplify the urgency for the need for approval of blood substitutes, with one pivotal trial that compared the only HBOC still available for human use, to erythrocytes.

While no product or trial is perfect, both studies that follow make serious attempts to address issues that had been unaddressed to date and, with hopeful regulatory approval in more countries, many lives may be able to be saved as a result of hemorrhage and surgical bleeding where blood is not an option or available.

## Consort abstract

As a landmark study was published just prior to the time CONSORT provided guidelines for structured abstracts^2^, the study abstract has been expanded and retrofitted to enable easier understanding with modern terminology (see Supplementary Table 1).

### Summary

The HEM-0115 Phase III prospective, randomized, single-blind trial included 688 subjects planned for orthopedic elective surgery, who were randomized at firsttransfusion decision to Hemopure (13 g/dL hemoglobin in 250 ml) or erythrocytes to treat surgery-related anemia (14). Hemopure has a p50 of 40 mm Hg compared to adult corpuscular hemoglobin of 27 mm Hg, so the oxyhemoglobin curve is shifted to the right to facilitate easier onloading and offloading of oxygen to the tissues.

Transfusion thresholds included [THb] of &lt;10.5 g/dL and a patient exhibiting at least one of the clinical signs: heart rate 100 bmp; SBP &lt;90 mm Hg or &lt;70% of preoperative screening value; electrocardiogram evidence of myocardial ischemia; metabolic acidosis (Base Deficit −4 or worse); acute blood loss &gt;7 mL/kg within 2 h or less. oliguria with urine output &lt;0.5 mL/kg/h ongoing for 2 h or more. The threshold would be considered high by today's care but was standard of care at the time the trial was undertaken (3). Additionally, transfusion thresholds included [THb] of 7 mL/kg within 2 h or less as an inclusion criterion, automatically (14).

Three hundred and fifty patients were randomized to a Hemopure infusion and 338 patients randomized to erythrocytes. Based on the cumulative clinical trial material units administered, the majority of Hemopure subjects received 5 or fewer 250 ml infusions with 18.7% receiving 6–10,250 ml infusions; one patient received 330 g or 11 infusions. By contrast, the erythrocyte subjects received 243 ± 9 g Hb on average, with 78.4% receiving ≥2 units (14).

Once treatment was initiated with a 2-unit (500 ml) loading dose of Hemopure, additional treatment was permitted for up to 6 days for a maximum of 10 units Hemopure (130 g hemoglobin) using the same criteria as for enrolment. The need for continuing oxygen transport beyond 10 units of Hemopure was met by crossing over Hemopure randomized subjects to erythrocytes. Transfusion avoidance was the primary outcome including a blinded assessment of safety. The designs of this Phase III study required that limits be placed regarding the volume of Hemopure infused. In severe hemorrhage, after limits of Hemopure infusions were met, patients were crossed over to receive erythrocytes. The clinical trial scenarios simulated circumstances where Hemopure was used until erythrocytes became available to replace moderate (≤ 3 units erythrocytes) blood loss for elective surgery (14).

#### Primary outcome

Among 350 HBOC-201 patients in the HEM-0115 trial, 96% avoided erythrocyte transfusion at Day 1, 70% at Day 7 and 59% at 6 weeks after surgery (the 95% confidence intervals were not presented). Low total Hb in combination with restricted subject activity was the reason most often documented for the first transfusion decision followed by tachycardia (100 beats/min). Achievement of the primary efficacy outcome, with subjects in this group receiving Hemopure, 59%, did not receive erythrocyte transfusions throughout the entire 6-week study period. Considering the intent to replace up to 6 units of erythrocytes by up to 10 units of HBOC-201, the actual rate of full blood avoidance was even higher since 317 (94%) of subjects in erythrocyte arm received 6 or less units. Administration of Hemopure resulted in an expected hematocrit reduction related to the acellular Hemopure solution infusions through treatment Day 5; in both cohorts, hematocrit values returned to normal at 6 weeks. An infusion of the loading dose of two units of Hemopure produced a 1.44 ± 0.03 g/dL increase in plasma hemoglobin and a 0.39 ± 0.06 g/dL increase in [THb]. Analysis of the total hemoglobin concentrations [THb] as a function of how many infusions received, revealed that subjects needing continuing Hemopure infusions, revealed a lower [THb] (p &lt; 10.5 g/dL) at a hemoglobin level of 8.85 ± 0.07 g/dL throughout the treatment period, excepting the loading 2-unit dose post-Hemopure total hemoglobin. HEM-0115 study subjects (n = 139), whose oxygen transport requirements were unmet by 10 units Hemopure, by protocol, were transfused erythrocytes (14).

#### Secondary outcomes

Examining the laboratory studies in the HEM-0115 study, the following laboratory values were not different between cohorts: albumin, alkaline phosphatase, total bilirubin, glucose, glutamyl transferase, lactate dehydrogenase and acid base-values. However, at follow up, an elevated total protein in the Hemopure group was noted, including an increase in aspartate aminotransferase and alanine aminotransferase. Lipase was increased in the Hemopure group in 5–11% of patients vs. 1–2% of patients in the erythrocyte group. Creatinine was increased 25% over baseline in 12 subjects (6%) from the Hemopure group compared with 3 subjects (2%) from the packed red blood cell group (14).

A summary of overall medical risk assessment for the intent to treat mandated by protocol, determined by blinded review of patient medical records and adverse events by treatment group, demonstrated the overall odds ratio for adverse events was 1.41–1.43 between groups (the 95% confidence intervals were not presented). Deaths were reported in10 subjects randomized to Hemopure and six in the patients randomized to PRBC (*p* =0.450). No deaths in either treatment group were categorized as associated with either treatment (14).

### Discussion and context

As discussed in Section Advances in second generation HBOCs and beyond, development of Hemopure started in the late 1980s and by mid-1990s had completed multiple clinical trials, under the FDA Phase I and II designation (12). My participation in the Phase II Cardiac trial of Hemopure (15), that was designed as a double-blind protocol and proved extremely difficult to blind the investigators (and subjects) as to which cohort they had been randomized into, helped dictate the single-blind protocol that was eventually designed and utilized for the Phase III.

This study and many to follow on Hemopure (16) have led to use in South Africa indicated for anemia and if blood is not an option or available and, in the US, and EU for Expanded Use/Compassionate use (3). Additional uses currently include preparation to conduct a trauma trial in South Africa, funded by the US Military^3^ as a perfusate in organ donor perfusion systems to lengthen the down time for organ transplants between harvest and implantation (37) and other novel indications (3).

The US FDA and EU regulatory authorities have yet to approve Hemopure for routine use as an oxygen therapeutic and may do so with positive results of the planned trauma trial. Niche uses will also likely be approved, especially when blood is not an option or available.

Discussion of these research findings by other investigators has highlighted that the imbalance in adverse events and serious adverse events are not the result of toxicity of the product but were likely a mix of subject pre-existing conditions, how management of clinical issues differed amongst practitioners and possible inadequate volumes of Hemopure infusions, in the face of continuing bleeding and low hemoglobin concentrations and hypoxemic tissues on one side of the equation and possible over infusion of Hemopure with congestive heart dysfunction on the other (38–40). Mackenzie et al. highlighted the challenges of conducting research in urgent/ emergency settings, leading to incomplete data, one of the challenges faced in this trial (41). Others highlight the benefits of blood substitutes as they are extensively scrubbed to remove infectious agents (including bovine encephalitis) and may be administered immediately when required in the field or ambulance avoiding the need of blood type and crossmatch procedures (42). As this trial was designed more than 20 years ago and was completed with enrolment in 2001, there are some significant shortcomings that may have been avoided had the CONSORT guidelines been published and adopted (see text footnote 2).

The first weakness is that attempting to compare an HBOC with a circulating plasma half-life of no longer than 24 h, with erythrocytes, depending on how fresh they are, which may circulate up to 30 days. However, this was the only head-to-head study against red blood cells as there is no comparator (crystalloids, colloids, plasma) that exists that is approved, due to lack of oxygen carrying capacity. This study did, in fact, demonstrate savings of erythrocytes, especially in the cohort of under 80-year-old subjects and patients in whom a hematinic effect could be demonstrated, with high reticulocyte counts and regeneration of autologous erythrocytes (43). Hemopure's breakdown is like any other hemoglobin and is, ultimately, turned to bilirubin but, in the process, there is a huge usable iron load released as well as globin proteins, often when the body may be starved due to medical therapy. This hematinic effect has been challenging to document yet, if it exists, might prove one of the most valuable attributes of a product as this.

Additionally, the transfusion thresholds or triggers for transfusion have been modified to around 7 g/dL (44) and not the 9–10 g/dL that were used in the trial. This could mean that the infusions of Hemopure were either unnecessary or provided other benefits as indicated above. Moreover, there may be some value in higher hemoglobin (and plasma and platelets) early in trauma or a surgical blood loss and may improve outcomes (45). Either way, the importance of the study is somewhat diminished by this and, were it to be repeated, it would need to be infused at hemoglobin concentrations <7 g/dL.

A comparison of [THb] and serious adverse events (SAEs) among HEM-0115 subjects given 10 units Hemopure (*n* = 211) and those given Hemopure plus erythrocytes showed that [THb] was significantly lower (&lt;10 Hemopure 250 ml infusions were identical to those receiving ≤ 3 units erythrocytes). Five deaths in the Hemopure arm occurred in patients older than 80 years compared with 1 in the erythrocyte group.

There was an equal number of deaths (*n* = 5) among patients under 80 years of age receiving either treatment. The HEM-0115 trial demonstrated that Hemopure with a hemoglobin concentration of 13 g/dL in comparison with erythrocytes provided management of hemorrhage of up to 3 units for elective orthopedic surgery without the use of erythrocytes or significant differences in mortality or serious adverse events. Weaknesses in this study could have been mitigated, had it been performed after the CONSORT guidelines (see text footnote 2) were promulgated. It would likely have questioned the direct comparison against erythrocytes and focused more on oxygen delivery, especially to the microcirculation, where the effect of oxygen delivery is required. Evaluating macrocirculatory issues, such as blood pressure and heart rate, amongst others, may be primitive, in terms of understanding the efficacy of an oxygen therapeutic, albeit important for resuscitation and survival. Also, the discrepancy between the Intent to Treat and those actually treated, which was due to situations where Hemopure was administered, did not yet meet inclusion criteria for Hemopure infusion as a protocol violation, would have been prevented from entering the trial from the start of infusion, not post hoc during data tabulation and analysis.

These findings demonstrate that Hemopure may be useful in the prehospital phase of trauma patient management when blood is unavailable. Also, Hemopure with hemoglobin concentration equivalent to whole blood may be infused safely to avoid up to 3 units of erythrocytes for elective surgery. Ensuring adequate oxygen delivery and coagulation factors are present are important considerations when administering Hemopure.

### Significance

This multicenter, single-blind, randomized, multinational study was one of the two largest clinical trials ever conducted with HBOC products (close to 700 subjects). The purpose of the study was whether use of Hemopure would reduce allogeneic red blood cells (PRBC) in orthopedic patients requiring blood transfusion. In subjects with moderate transfusion needs, Hemopure was shown to significantly reduce PRBC use without increase in adverse effects. However, in patients with higher transfusion needs, Hemopure failed to correct the anemic condition due, in part, to unbalanced study design and inherent limitations of Hemopure. Considering the relatively more dilute Hb concentration, HBOC would inherently require more volume than erythrocytes. Therefore, in high demand subjects for comparable effect, larger volumes of HBOC were required that might have led to volume overload and higher incidences of adverse effects. This study taught the PI a critically important lesson regarding fluid resuscitation and risk of volume overload, a key need for formulating a small volume, multifunctional, resuscitation fluid.

### Conclusion of trial

Despite its successful ability to decrease the likelihood of transfusion in nearly 50% of the subjects studied, the FDA has not yet approved Hemopure; however, the FDA has allowed its use under expanded access and compassionate use (3). It may be used in situations in which all other options have been exhausted and the patient is experiencing severe and dangerous anemia and is approved for human use if blood is unavailable or not an option in South Africa and other countries.

### Summary of review

This review has attempted to describe a major morbidity and mortality of hemorrhagic shock, that is either inadequately treated or unable to be treated at the site of injury due to blood not being available or not an option and, in the perioperative setting, where expected blood loss is managed with erythrocytes and clotting factors but due to the inherent risks of allogeneic blood, may worsen outcomes.

Hemoglobin-based oxygen carriers may offer a solution to acute anemia, whatever the etiology and provide rapid onset oxygenation to tissues even without pulsatile blood flow. Doing so may improve the statistics of mortality of hemorrhage, which may account for between 1.9 and 5 million lives annually, dwarfing even devastating deaths due to the pandemic with COVID-19.

Therefore, continued work on HBOCs is warranted and, despite multiple setbacks with trials and products, it makes sense that further work be funded and continued, on the most promising products. The question of whether or not to create HBOCs with high or low p50's has been raised, and while Hemopure has shifted the oxyhemoglobin curve to the right, a number of newer products have been developed with lower p50's, analogous to fetal hemoglobin (p50 = 19 mm Hg), and even lower, with the concept being that only severely hypoxemic hemoglobin will load and offload oxygen, providing only to tissues severely depleted; however, no comparative studies have been performed to demonstrate which is more effective (46).

Hemopure may be one of those HBOCs, given its approval for human use since 2001 in South Africa and multiple trials in humans and continued use that demonstrate reasonable safety and efficacy. To compare this, or any product for that matter, against blood, which has never undergone safety or efficacy trials, may be meaningless; far more important is to find those niches where survival is improved while maintaining safety. This work has attempted to do just that: demonstrate reasonable safety and efficacy despite adverse events that all pharmacotherapies have and accept those with the understanding that no drug is side-effect free which would be an unlikely expectation. The challenge is to accept the side-effect profile and modify usage and indication so that benefits may outweigh risks, the challenge upon which all life is predicated.

## Author contributions

The author confirms being the sole contributor of this work and has approved it for publication.

## Conflict of interest

The author declares that the research was conducted in the absence of any commercial or financial relationships that could be construed as a potential conflict of interest.

## Publisher's note

All claims expressed in this article are solely those of the authors and do not necessarily represent those of their affiliated organizations, or those of the publisher, the editors and the reviewers. Any product that may be evaluated in this article, or claim that may be made by its manufacturer, is not guaranteed or endorsed by the publisher.

## Footnotes

^1^https://pathways.nice.org.uk/pathways/trauma (accessed January 15, 2021).

^2^http://www.consort-statement.org/extensions/overview/abstracts (accessed January 16, 2021).

^3^https://www.dvidshub.net/news/printable/311421 (accessed January 16, 2021).
