# Supplementary material for: Blood substitutes: Basic science, translational studies and clinical trials
Source: Front Med Technol. 2022 Aug 18;4:989829. doi: 10.3389/fmedt.2022.989829 (PMC9433579; doi:10.3389/fmedt.2022.989829)
Supplement: Supplementary file 1 [file Table_1.DOCX]

Table. CONSORT Abstract.

Format adapted from Reference 63, Content adapted from Reference 15. Jahr et al, JTrauma 2008.

| Item | Description |  |
| --- | --- | --- |
| Title | HBOC-201 as an Alternative to Blood Transfusion: Efficacy and Safety Evaluation in a Multicenter Phase III Trial in Elective Orthopedic Surgery |  |
| Authors * | Jonathan S. Jahr, MD, Colin Mackenzie, MD, L. Bruce Pearce, PhD, Arkadiy Pitman, MS, and A. Gerson Greenburg, MD, PhD |  |
| Trial design | Randomized, single-blind (subjects), parallel-group, multicenter, multinational, superiority trial. |  |
| Methods |  |  |
| Participants | Patients who provided informed consent, 18 years old and older, scheduled for elective and semi-urgent orthopedic surgery and with expected significant blood loss and likely need for transfusion |  |
| Interventions | Randomization at time of transfusion to either packed red blood cells or Hemopure, with repeated doses, up to 10 units of Hemopure and then crossing over to red blood cells if needed |  |
| Objective | The ability of Hemopure to safely reduce and/or eliminate perioperative transfusion was studied in orthopedic surgery patients.  Elimination of packed red blood cell transfusions in subjects receiving Hemopure in more than 35% subjects. |  |
| Outcome | Primary efficacy endpoint was transfusion avoidance. Primary safety endpoint was adverse events and serious adverse events. |  |
| Randomization | Allocation concealed, secure electronic randomization (1:1 ratio) |  |
| Blinding (masking) | Subjects blinded to which group they were assigned, not investigators or those collecting data on trial outcomes. |  |
| Results |  |  |
| Numbers randomized | Six hundred eighty-eight patients were randomized to treatment with Hemopure (H, n = 350) or packed red blood cells (R, n = 338) at the first transfusion decision. |  |
| Recruitment | 1999-2002. Sites in US, UK, EU and South Africa |  |
| Numbers analyzed | The primary analysis included 350 and 338 participants in the Hemopure and packed red blood cells groups, respectively (intention to treat group). |  |
| Outcome | A total of 59% of patients in the H arm avoided PRBC transfusion, exceeding the pre-defined superiority of 35% by almost double |  |
| Harms | Adverse events (8.5 vs. 5.9) per patient, and serious adverse events (SAEs) (0.35 vs. 0.25) per patient were higher in the H *versus* R arms (p < 0.001 and p < 0.01). |  |
| Sub-group analyses | The between arms (H vs. R) safety analysis was  unfavorable and likely related to patient age, volume overload, and undertreatment and was isolated to patients that could not be managed by HBOC-201 alone. However, patients younger than 80 years old with moderate clinical need may safely avoid transfusion when treated with up to 10 units of Hemopure. |  |
| Conclusions | Hemopure eliminated transfusion in the majority of subjects |  |
| Trial registration | NCT00301535 Countries: US FDA, EU, UK, South Africa |  |
| Funding | Manufacturer funded, US FDA Approved Phase III Trial |  |
